# Investigation of *Campylobacter fetus* in breeding bulls of private farms in Bangladesh

**DOI:** 10.1002/vms3.831

**Published:** 2022-07-11

**Authors:** Nazmul Hoque, Sk Shaheenur Islam, Md. Jahidul Islam Saddam, Md. Rafikuzzaman, Mahmudul Hasan Sikder, David M. Castellan, S. M. Lutful Kabir

**Affiliations:** ^1^ Department of Microbiology and Hygiene Bangladesh Agricultural University Mymensingh Bangladesh; ^2^ Department of Pharmacology Bangladesh Agricultural University Mymensingh Bangladesh; ^3^ Epidemiology Institute for Infectious Animal Diseases Texas A&M University Texas USA

**Keywords:** antimicrobial resistance, *Campylobacter fetus*, breeding bull, occurrence, risk factors

## Abstract

**Background:**

Bovine genital campylobacteriosis (BGC) is a venereal disease caused by *Campylobacter fetus* that has a negative impact on animal reproduction. The bull is considered to be a symptomless carrier that spreads the disease agent to breeding cows, causing infertility and sporadic abortion.

**Aim:**

The study aims to estimate the prevalence, identify risk factors of *Campylobacter fetus* (*C. fetus*) infection and antimicrobial resistance pattern of the *C. fetus* isolates.

**Method:**

A cross‐sectional survey was conducted in Mymensingh district of Bangladesh. Bull smegma samples (single sample from each bull) were collected from 300 bulls from four farms and tested via culture, biochemical identification and finally 16S rRNA and *cdtA* gene‐based molecular assays (PCR) for herd and animal‐level prevalence estimation. Herd‐ and animal‐level data on risk factors were collected from the farmers using a pretested questionnaire and analysed by univariable and multivariable logistic regression models with a *p* value of <0.05 was taken statistically significant for both analyses.

**Results:**

Among the surveyed farms, 75% (95% CI: 19.4%–99.4%) were confirmed to have bulls infected with *Campylobacter fetus* at herd level. However, animal‐level occurrence of *C. fetus* was estimated to be 8.7% (26/300) (95% CI: 5.7%–12.4%). Natural service increases the odds of campylobacteriosis 38.18 times (95% CI: 13.89–104.94) in comparison to artificial insemination for C*. fetus* infection in bulls. Significantly, half of the isolates (50%, *n* = 13) were identified to be multidrug resistant (MDR) for three to five antimicrobial agents.

**Conclusion:**

This study highlights the need to develop official guidelines for *C. fetus* control and prevention in Bangladesh including mandatory artificial insemination in reproductive cows and heifers, routine screening of breeding bulls for *C. fetus* free status.

## 1 INTRODUCTION

*Campylobacter fetus* (*C. fetus*) is a spiral, Gram‐negative, motile bacterium, which causes campylobacteriosis (Hoffer, 1981). *C. fetus* is classified into two subspecies, namely, *C. fetus* subsp. *fetus* (*Cff*) and *C. fetus* subsp. *venerealis* (*Cfv*), both of them are associated with the poor reproductive health of ruminants (Cagnoli et al., 2020). Generally, *C. fetus* subspecies *venerealis* (*Cfv*) is reported to be the causative agent of venereal diseases of cattle, for example, bovine genital campylobacteriosis (BGC), while the bacterial infection eventually lead to lowered reproductive performance (repeat breeders), due to more commonly premature embryonic death (World Organization for Animal Health, 2018). However, *C. fetus* subsp. *fetus (Cff)* and *C. jejuni* are also associated with lowered fertility and abortion in cattle (Vargas et al., 2003). *Campylobacter* spp. may be present in male external genitalia (penis) including in the preputial mucosa, and female cervix, uterus, oviducts and vaginal mucosa (Garcia et al., 1983; Spósito Filha & Oliveira, 2009).

Male cattle are frequently symptomless; however, females show various degrees of clinical signs, including irregular oestrus, vaginitis, endometritis, cervicitis, early embryonic deaths or even abortion and temporary infertility (BonDurant, 2005; Dawson, 1986; Rae, 1989). Analysis of samples from reproductive organs of both male (preputial smegma) and female animals (vaginal mucus) including internal organs of the aborted fetus are used to confirm the presence of *C. fetus* infection in cattle herds (Campero et al., 2005; Garcia et al., 1983; Penner, 1988). Interestingly, infection in breeding bulls does not hamper sperm quality or even libido (Eaglesome & Garcia, 1992; Van Bergen et al., 2005). Therefore, bulls should be considered using a targeted approach for herd health screening and for epidemiological studies to build strategies for prevention and control.

Bovine genital campylobacteriosis is transmitted primarily through natural mating in cattle breeding program; however, infection is also spread via infected bull semen used in artificial insemination (AI) and/contaminated equipment (Modolo et al., 2000). Transmitted between female cattle is less likely since the disease usually spreads through natural mating/coitus (Clark, 1971; Hoffer, 1981); however, transmission between bulls may occur during mounting when distance between breeding bulls is inadequate, and several bulls are kept in the same house (Taylor, 2002). Infection with BCG does not hamper conception; however, it usually culminates with early embryonic loss, and therefore, delayed oestrus, while abortions in the infected female are most frequently detected between 4 and 6 months of pregnancy period. The disease is generally self‐limiting in females, and the acquired immunity usually persists for several years (Mshelia et al., 2007; Taylor, 2002). The bulls can also be exposed to *Campylobacter fetus* infection from the infected cows during natural mating. The pathogens grow in the crypts of the penis and preputial sac; however, they are not able to invade into the deeper tissue mass. A bull may either be infected temporarily or over a period year. Therefore, the bulls can act as a reservoir and disseminate this pathogen through coitus (Eaglesome & Garcia, 1992). Nevertheless, long‐lasting infections are observed in aged bulls due to presence of deeper penile and preputial crypts where the organisms reside. Bulls, which contract infection from diseased cows during breeding, do not develop convalescent immunity, and therefore, mostly remain susceptible to reinfection.

Growing demand for animal origin food especially red meat is estimated to be increased twofold by 2050 in small and middle income countries (Agus & Widi, 2018).To mitigate such demand numerous initiatives have been taken by the government through intervention of many projects and programs for the development of livestock sectors since several decades ago. These activities have directed to improve the productivity of native cattle for genetic improvements by artificial insemination (Department of Livestock Sevices, 2007). There are approximately 24.39 million cattle, 1.49 million buffaloes, 26.4 million goats, and 3.6 million sheep in Bangladesh in 2019–2020 (Department of Livestock Sevices, 2020). At present, among the total cattle population, 15% are high yielding crossbred stocks (Hamid et al., 2017) of Holstein Friesian, Sindhi, Sahiwal with a small proportion of Jersey breed (Miazi et al., 2007, Islam et al., 2020). As the majority of the female cattle in Bangladesh are not taken under the artificial insemination program which may increase the likelihood of transmission *C. fetus* infection in cows through natural mating from the infected bulls (Clark, 1971; Hoffer, 1981).

As a part of *C. fetus* surveillance, limited screening of breeding bulls from government farms in Bangladesh is being conducted, but surveys are not carried out of private breeding bulls. However, there is no published report on the occurrence of *C. fetus* in breeding bulls in Bangladesh. Nevertheless, a study confirmed 0.3% *C. fetus* prevalence in cattle of selected dairy farms of Dhaka and Mymensingh districts of Bangladesh via faecal sample evaluation (Hoque, 2021). Bangladesh sends official notifications regularly to the World Organization for Animal Health (OIE) on presence of BGC as per standard criteria (World Organization for Animal Health, 2021). This cross‐sectional study is conducted to investigate the occurrence, molecular traits, antimicrobial resistance and risk factors of *C. fetus* infection in breeding bulls from private farms in of Bangladesh. This is the first study in Bangladesh to assess the presence of *C. fetus* infection in breeding bulls through molecular detection and risk factor assessment that will assist in formulating strategies to prevent and control measures for reproductive health promotion of cattle.

## 2 MATERIALS AND METHODS

### 2.1 Study design and location

A cross‐sectional survey was conducted during January 2019 to June 2020 in Mymensingh district, a promising cattle rearing zone of Bangladesh with an estimated 923,000 heads of cattle (Department of Livestock Sevices, 2017), of which 15% are crossbred farmed cattle (Hamid et al., 2017).

### 2.2 Sample size and surveyed farms

**FIGURE 1:**
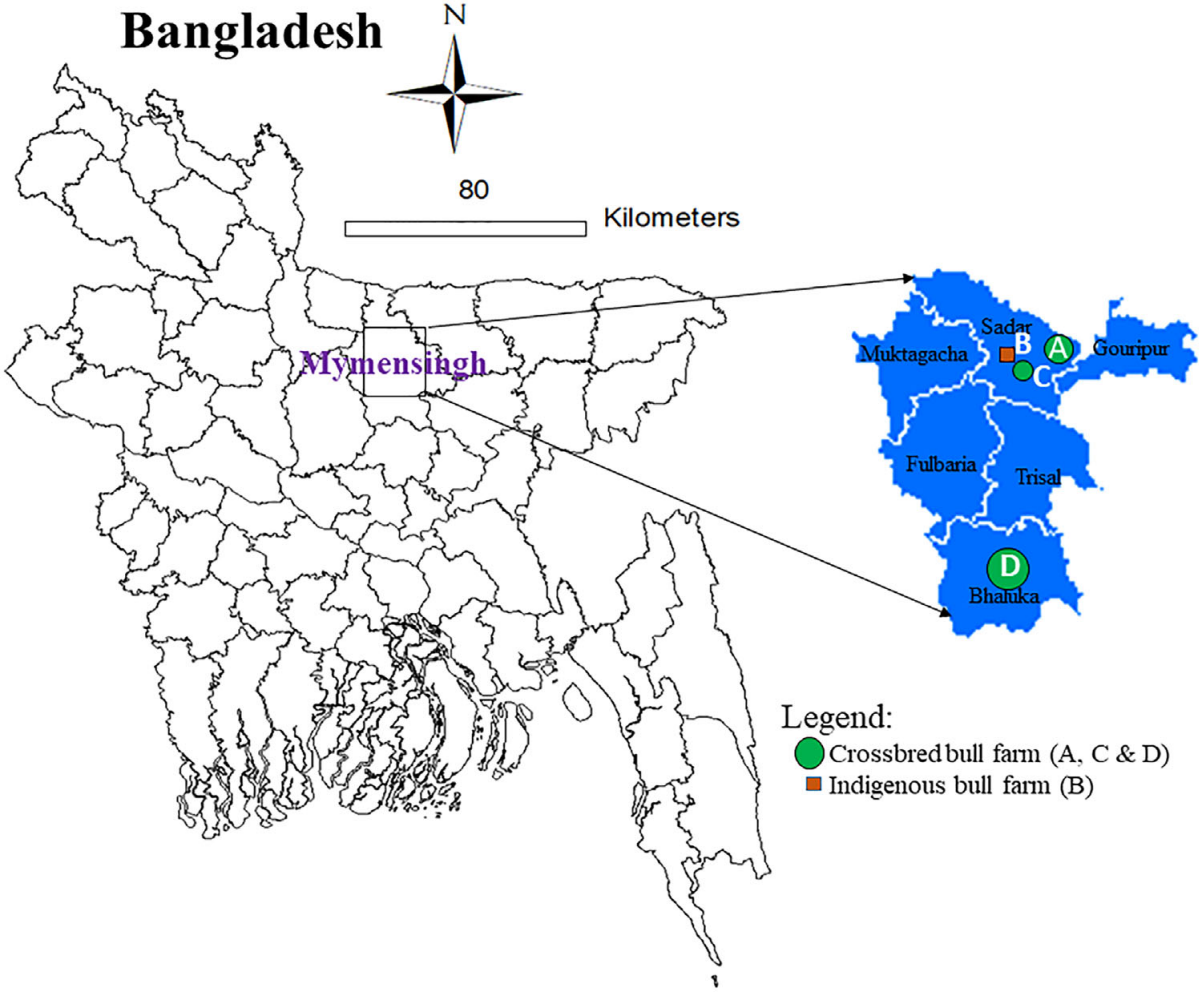
Location of study farms in Mymensingh district of Bangladesh, 4 breeding farms were included under this study, of which 3 farms (1 indigenous and 2 crossbred bull farms) from Mymensingh Sadar subdistrict and 1 bull farm from Bhaluka subdistrict of Mymensingh district were included under this study.

The Department of Livestock Services (DLS), Bangladesh, covers nearly 55% of artificial insemination (AI) in national breedable cows (Department of Livestock Services, 2021). Apart from government initiatives, six registered private commercial breeding bull farms in Bangladesh are also producing frozen semen for AI to support to the marginal communities in rural areas targeted to better quality cattle breeds. Two big registered private commercial breeding bull farm and another two (one indigenous and one crossbred) bull farms with 410 bulls were considered as study population under this study. Two commercial breeding farms have 310 bulls (those are producing semen for AI), of which 258 bulls and remaining 42 bulls were taken from another two farms; in total 300 bulls were taken randomly for this study. Bull of the Holstein Friesian, Sahiwal, Jersey and Red Chittagong breeds are usually used for the production of frozen semen in private and government breeding farms. So, we think this sample will represent the breeding bull populations in Bangladesh (Figure 1).

### 2.3 Data collection

The data and samples were collected by an expert veterinarian and one veterinary paraprofessional. A semi‐structured questionnaire was used for collection data from farmers/farm managers during data collection and subsequent animal sampling (Table S1). The questionnaire was designed to capture variables related to (i) herd‐level determinants (10 questions) and (ii) animal‐level determinants (7 questions). Apart from field interview of the herd managers, some herd‐level data were also obtained and/or validated through a transect walking method (like breed, husbandry type, presence of biogas plant, biosecurity status, etc.). The responses of the questionnaire were coded and stored in Excel data sheet for descriptive and inferential analyses.

### 2.4 Sampling procedure

Since bulls are usually asymptomatic carriers of *C. fetus* associated with BGC infections in the endemic herd (BonDurant, 2005), samples were collected from the preputial crypts of the penis of the bulls. The smegma samples were obtained by scraping from the preputial and penile mucosa using a disposable plastic scraper in 50 mL falcon tube (Tedesco et al., 1977), afterwards, rinsed in 20–30 mL of phosphate buffered saline (PBS) following method established previously (Mordasini et al., 2006).

**FIGURE 2:**
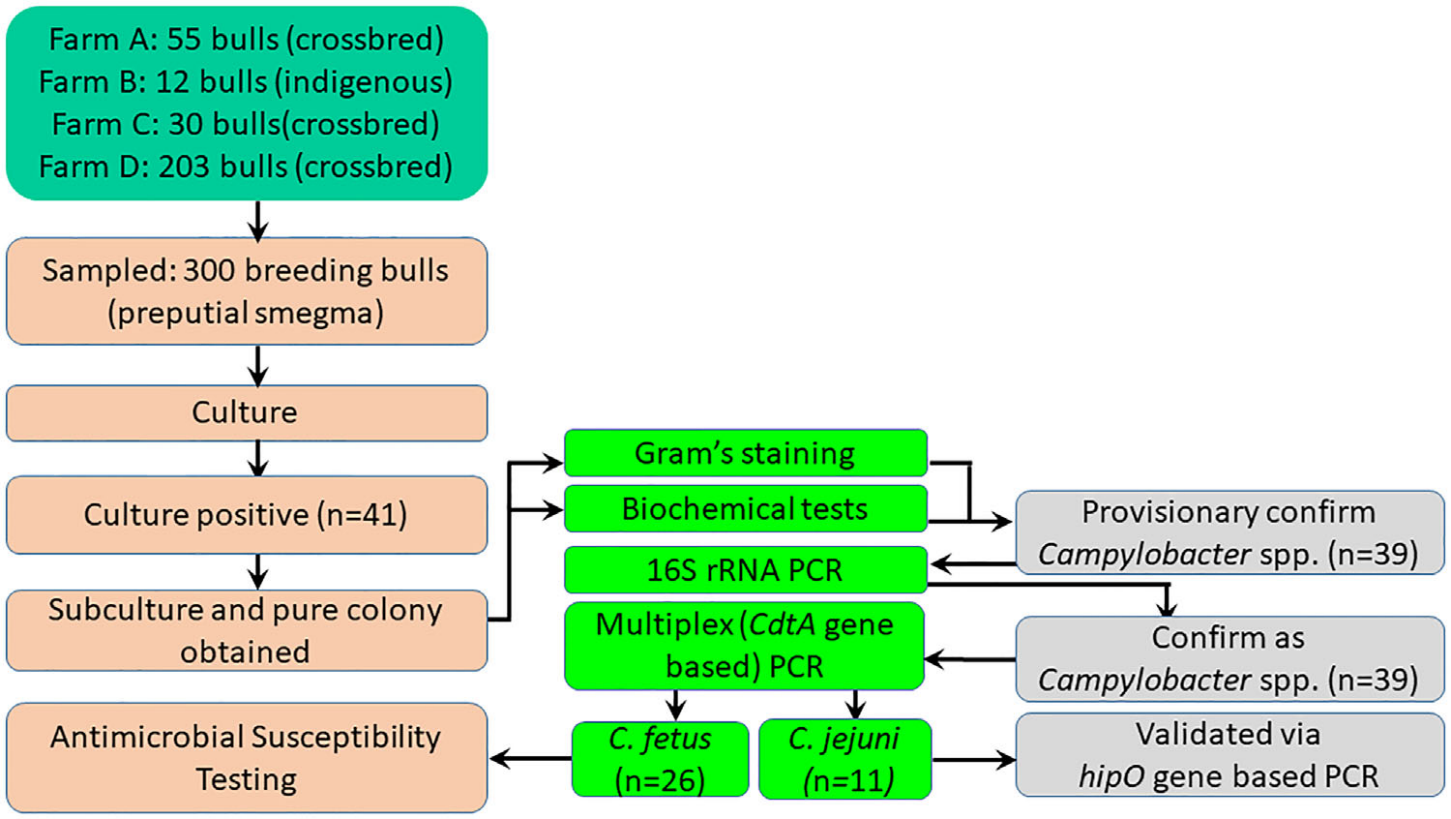
Schematic diagram of sample collection and evaluation workflow.

Aseptic procedures were maintained during the collection of the samples, which were transported, maintaining a cold chain at 4–6°C, to the laboratory and processed within 4–6 h of collection. A schematic diagram of sample collection and testing workflow is presented in Figure 2.

### 2.5 Laboratory analysis

#### 2.5.1 Culture‐based methods and biochemical tests

Samples were subjected to a filtration method using cellulose filter with a pore size 0.65 μm (Biotech, Göttingen, Germany) as described previously (Bolton et al., 1988), with minor adjustment. Briefly, 100 μL of each sample was spread on the filters that were kept on the surface of blood agar base (no. 2) (HiMedia, Mumbai, India) with supplementation of 5% sheep blood and growth supplement (HiMedia, Mumbai, India) to isolate *C. fetus* (World Organization for Animal Health, 2021), and then allowed to stand at room temperature for 30 min. Afterwards, the filters were removed and the media were incubated at 37°C for 48 h under microaerophilic conditions, that is, 5–10% oxygen, 5–10% carbon dioxide and 5–9% hydrogen for optimal growth of the campylobacters (Vandamme, 2000), using AnaeroPouch^®^‐MicroAero (Mitsubishi Gas Chemical Co., Inc., Tokyo, Japan). Colonies having the typical morphological traits of *Campylobacter* spp., (i.e., grey, flat and irregularly spreading colonies) grown on the incubated media were selected. The Gram staining was performed on the selected colonies and the Gram‐negative curved‐shaped cells, observed under a light microscope, were considered as presumptive *Campylobacter* spp. A single and pure colony was obtained after subculture of the presumptive *Campylobacter* colonies using blood agar media. A tentative confirmation of the isolates was achieved based on growth characteristics and biochemical tests, including catalase test, oxidase test, hippurate hydrolysis test, nitrate reduction test, indoxyl acetate test and 1% glycerine test as per standard protocols (Foster et al., 2004; Nachamkin, 2003; Swai et al., 2005).

#### 2.5.2 Polymerase chain reaction (PCR)

The tentative *Campylobacter* isolates were subjected to a genus‐specific PCR assay to validate the presence of *Campylobacter* spp. A pure culture of tentative *Campylobacter* spp. was used for DNA extraction via boiling method (Hoshino et al., 1998). The amplification of the 16SrRNA gene using oligonucleotide primers was done following previously established method (Samosornsuk et al., 2007) as shown in the Table 1. Finally, a multiplex PCR assay targeting *cdtA* gene was done as serial testing for molecular identification of different species of *Campylobacter* (i.e., *C. jejuni*, *C. coli* and *C. fetus*) according to the method of Asakura et al. (2008) (Table 1). However, after confirmation by this multiplex PCR assay, the isolates were further screened using *hipO* gene‐based PCR to validate the identification of *C. jejuni* (Linton et al., 1997) (Table 1 and Figure S1).

**TABLE 1:** List of primers and thermal condition used for molecular detection of *Campylobacter* species

Target gene	Primers	Sequence (5´–3´)	Amplicon size (bp)	PCR condition for 30 cycles	Reference
16S rRNA	16S9F 16S1540R	GAGTTTGATCCTGGCTC AAGGAGGTGATCCAGCC	1530	(1) Denaturation at 94°C for 30 s, (2) annealing at 47°C for 30 s and (3) extension at 72°C for 90 s	(Samosornsuk et al., 2007)
*CjcdtA*	Cj‐CdtAU2 Cj‐CdtAR2	AGGACTTGAACCTACTTTTC AGGTGGAGTAGTTAAAAACC	631	(1) Denaturation at 94°C for 30 s, (2) annealing at 53°C for 30 s and (3) extension at 72°C for 90 s	(Asakura et al., 2008)
*CccdtA*	Cc‐CdtAU1 Cc‐CdtAR1	ATTGCCAAGGCTAAAATCTC GATAAAGTCTCCAAAACTGC	329		
*CfcdtA*	Cf‐CdtCU2 Cf‐CdtCR2	AACGACAAATGTAAGCACTC TATTTATGCAAGTCGTGCGA	489		
*hipO gene*	HIP400F HIP1134R	GAAGAGGGTTTGGGTGGTG AGCTAGCTTCGCATAATAACTTG	*735*	(1) Denaturation at 94°C for 30 s, (2) annealing at 55°C for 30 s and (3) extension at 72°C for 45 s	(Linton et al., 1997)

In the multiplex PCR assay, DNA templates of *C. jejuni* ATCC 33560, *C. coli* ATCC 33559 and *C. fetus* ATCC 27374 strains were used as positive controls, whereas *Escherichia coli* ATCC 25922 strain was used as a negative control. PCR products were subjected to a gel electrophoresis (1.5% agarose, Invitrogen, Carlsbad, CA, USA), and subsequently, stained with ethidium bromide (0.5 g/mL) and de‐stained with distilled water, for 10 min each. Subsequently, the gel images were captured using a UV transilluminator (Biometra, Göttingen, Germany).

### 2.6 Antimicrobial susceptibility testing

All of the isolated strains of *C. fetus* were checked for antimicrobial susceptibility using the disk diffusion method (Luangtongkum et al., 2007) against eight commonly used antimicrobials, namely amoxicillin (30 μg), ciprofloxacin (5 μg), erythromycin (30 μg), azithromycin (30 μg), tetracycline (30 μg), gentamicin (10 μg), streptomycin (10 μg) and norfloxacin (10 μg) (HiMedia, Mumbai, India). The inhibition zones of growth were compared with the zone diameter as per standards suggested by the Clinical and Laboratory Standard Institute (2016), and thereby, determined as resistant (R), intermediate resistant (I) or susceptible (S) to the antimicrobial agents. *E. coli* strain ATCC 25922 was used as a quality control organism. The results of antimicrobial susceptibility tests were confirmed by conducting at least two replicates of the disk diffusion assay.

### 2.7 Statistical analysis

Data generated through field interview and laboratory testing were captured in Microsoft Excel 2010 (MS Excel) datasheet, checked for data quality and coded for systematic comparison and exported to STATA 13 (USA, StataCrop, 4905, Lakeway Drive, College station, Texas 77845, USA) and R 3.6.0 (Team, 2019) for further analysis.

Data on demographic and risk factors were summarised using descriptive statistics. In this evaluation, mean and standard deviation (SD) were estimated for continuous variables; however, proportions and frequency distributions were calculated for categorical variables. All continuous variables like age of the animal, body weight were categorised before inclusion for logistic regression analysis.

#### 2.7.1 Univariable mixed‐effect logistic regression analyses

Univariable mixed effect logistic regression analyses were performed by including herd as a random intercepts (Bates et al., 2016). In this evaluation, *C. fetus* infection status as the response and each risk indicators in turn as an explanatory variable in this model. Collinearity among the variables was evaluated by Cramer's phi‐prime statistic (R package ‘vcd’, ‘assocstats’ function (Meyer et al., 2017). When Cramer's phi‐prime statistic was >0.70, a pair of variables was considered collinear (Rahman et al., 2017). The herd‐level data were not suitable for inferential interpretation since the number of herds were very small (*n* = 4).

## 3 RESULTS

### 3.1 Descriptive statistics

The survey was conducted on a sample group of 300 bulls from Mymensingh district of Bangladesh (Figure 1). Among the four bull farms, the median number of bulls in each herd was 43 (12‐203). Of the total population of bulls, 96% (*n* = 288) were high‐yielding crossbred variety, comprising mostly of Holstein Frisian crossbred, and some were Sahiwal/Jersey crossbred. All the farms were established for at least 5 years. Three of the farms under intensive farming with good biosecurity standards. In two of the study farms, feeding of bulls included the ready‐made commercial feed (TMR). Half (*n* = 2, 50%) of the study farms practiced commercial artificial insemination program (Table 2). All the farms had concrete floors. A single farm had a biogas plant to utilise cattle faeces for gas production. The annual screening of breeding bulls for *C. fetus* infection was carried out once a year in two of the farms.

**TABLE 2:** Herd composition, management practices and herd‐level occurrence of *C. fetus* in four breeding farms in Mymensingh district of Bangladesh

Factor	Category	Frequency (%)	Occurrence of *C. fetus* within herd[number of herds (%)]
Breed	Crossbred	3 (75.0)	2 (66.7)
	Indigenous	1 (25.0)	1 (100.0)
Herd size	10–20	1 (25.0)	1 (100.0)
	20–50	1 (25.0)	1(100.0)
	>50	2 (50.0)	1 (50.0)
Type of husbandry	Intensive	3 (75.0)	2 (66.7)
	Extensive/semi‐intensive	1 (25.0)	1 (100.0)
Biosecurity status	Good	3 (75.0)	2 (66.7)
	Bad	1 (25.0)	1 (100.0)
Rearing other animals (sheep, goat, poultry)	Yes	2 (50.0)	2 (100.0)
	No	2 (50.0)	1 (50.0)
Breeding program	Natural	2 (50.0)	2 (100.0)
	Artificial	2 (50.0)	1 (50.0)
Presence of biogas plant	Yes	1 (25.0)	1 (100.0)
	No	3 (75.0)	2 (66.7)
Routine screening of *C. fetus* in the herd	Yes	2 (50.0)	1 (50.0)
	No	2 (50.0)	2 (100.0)
Feed	Commercial (TMR)	2 (50.0)	1 (50.0)
	Ready‐made feed	2 (50.0)	2 (100.0)

### 3.2 Herd‐ and animal‐level prevalence

Among the four farms, three were found to be positive (least one positive animal on a farm) through culture, biochemical tests and finally molecular assays, hence, herd‐ and farm‐level occurrence of *C. fetus* infection was documented as 75% (95% CI: 19.4‐99.4%) (Table 3). A clear‐cut influence of any factor concerning herd composition and management practices on the farm‐level occurrence of *C. fetus* infection in bull samples was not discernible. Of the 300 samples (a single sample from each bull), 26 were positive for *C. fetus* infection that represents an apparent animal‐level prevalence of 8.7% (95% CI: 5.7‐12.4%) (Table 3). However, this study additionally confirmed the occurrence of *C. jejuni* in 11 samples, an apparent prevalence of 3.7% (95% CI: 1.8‐6.5%) of the bulls smegma samples (Figure 2).

**TABLE 3:** Animal‐level prevalence of C*. fetus* in four breeding bull farms in Mymensingh district, Bangladesh

Herd ID	Animal/sample tested (*N*)	Culture* positive (*n*)	Prevalence (%)95% CI	PCR** positive (*n*)	Prevalence (%)95% CI
1	55	0	0 (0–6.5)	0	0 (0–6.5)
2	12	8	66.7 (34.9–90.1)	5	41.7 (15.2–72.3)
3	30	22	73.3 (54.1–87.7)	15	50.0 (31.3–68.7)
4	203	9	4.4 (2.0–8.2)	6	3.0 (1.1–6.3)
Total	300	39	13.0 (9.4–17.3)	26	8.7 (5.7–12.4)

Abbreviation: CI, confidence interval.

* Culture and biochemical tests (*Campylobacter* spp.).

** 16S rRNA gene‐based genus‐specific PCR followed by multiplex *cdtA* and *hipO* gene‐based PCR (*C. fetus*).

### 3.3 Evaluation of animal‐level risk factors

Seven variables were included in the univariable mixed‐effects logistic regression analysis. Among the variables, breeding program was found to be significantly associated with animal‐level *C. fetus* infection‐positive status. Natural service increases the odds of campylobacteriosis 38.18 times (95% CI: 13.89‐104.94) in comparison to artificial insemination in breeding bulls (Table 4). Since a single variable was found to be significant univariable mixed‐effects logistic regression analyses; therefore, building multivariable mixed‐effect logistic regression model was not possible.

**TABLE 4:** Univariable analysis of potential risk factor for animal‐level infection with *C. fetus*

Variable	Category	Positive (%)	Estimate	Standard error	Odds ratio (95% CI)	*p* Value
Age of the animal	>3 years (*n* = 93)	8 (8.6)	–0.4652	0.5540	Reference	0.401
	≤3 years (*n* = 207)	18 (8.7)	‐	‐	NE	
Breed	Indigenous (*n* = 12)	5 (41.7)	2.564	2.373	NE	0.27
	Crossbred (*n* = 288)	21 (7.3)	‐	‐	Reference	
Source of animal	Farm (*n* = 168)	17 (10.1)	0.4252	0.6381	NE	0.51
	Bought (*n* = 132)	9 (6.8)	‐	‐	Reference	
Health condition	Bad (*n* = 10)	3 (30)	1.144	1.028	NE	0.27
	Good (*n* = 290)	23(7.9)	‐	‐	Reference	
Bogy weight	Up to 300 kg (*n* = 161)	19 (11.8)	0.4680	0.6728	NE	0.48
	Above 300 kg (*n* = 139)	7 (5.0)	‐	‐	Reference	
Breeding program	Artificial insemination (AI) (*n* = 258)	6 (2.3)	‐	‐	Reference	
	Natural service (*n* = 42)	20 (47.6)	3.64	0.52	38.18 (13.89–104.94)	<0.001
Bad behaviour (mounting)	Yes (*n* = 43)	8 (18.6)	0.04939	0.58751	NE	0.93
	No (2 = 257)	18 (7.0)	‐	‐	Reference	

Abbreviations: NE, not estimated; CI, confidence interval.

### 3.4 Antibiogram

#### 3.4.1 Antimicrobial susceptibility status

**FIGURE 3:**
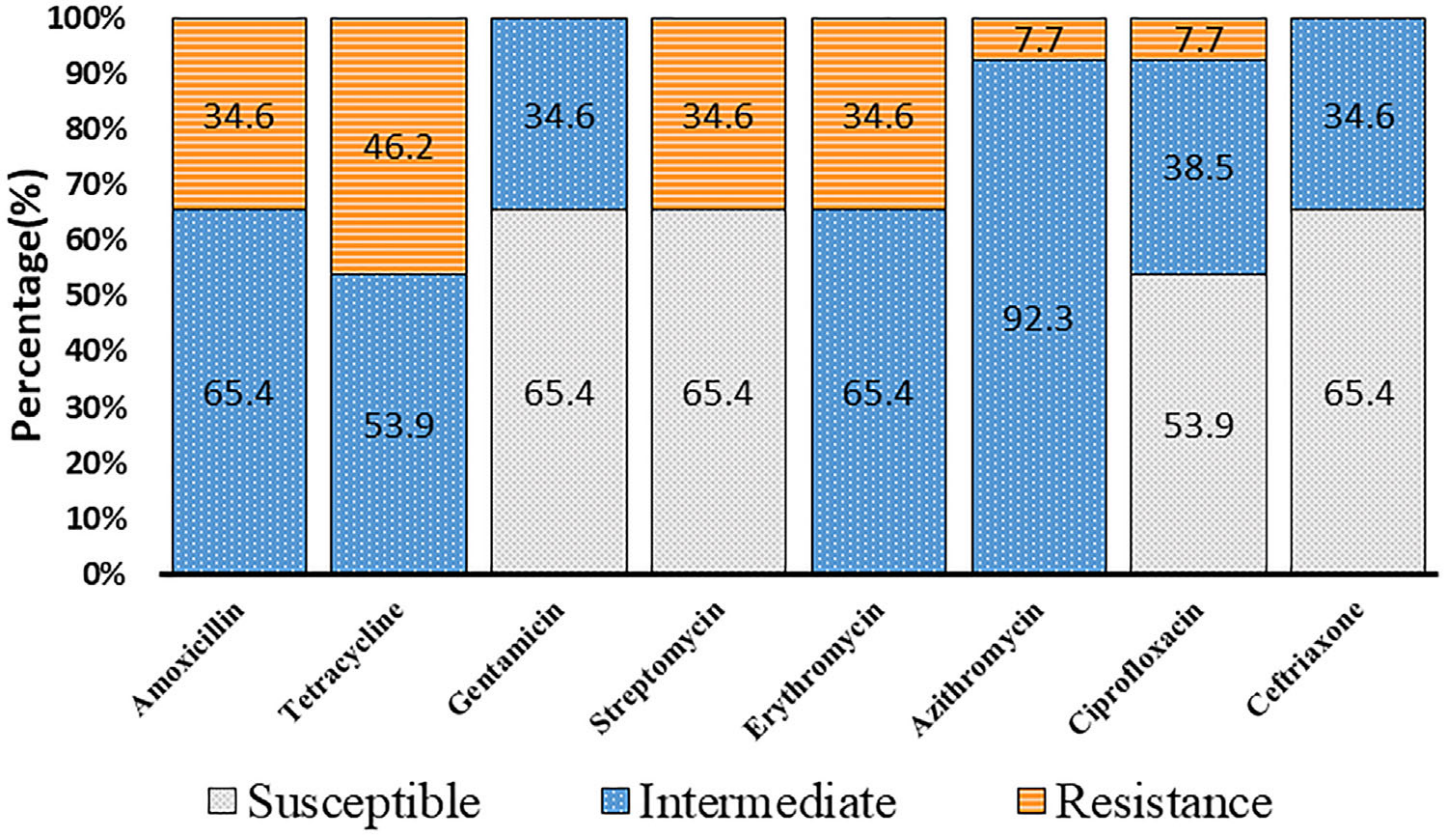
Antimicrobial susceptibility profile of *C. fetus* (*n =* 26) against commercially available antimicrobial agents at standard doses. The antimicrobial agents included amoxicillin (30 μg), ciprofloxacin (5 μg), erythromycin (30 μg), azithromycin (30 μg), tetracycline (30 μg), gentamicin (10 μg), streptomycin (10 μg) and norfloxacin (10 μg). The *C. fetus* strains were categorised based on three types of resistance status: susceptible, intermediate and fully resistant, in accordance to Clinical and Laboratory Standards Institute (CLSI, 2016).

Of 26 isolates of *C. fetus*, 65.4% (*n* = 17) were found to be susceptible to gentamicin, streptomycin, and ceftriaxone, while, 53.9% (*n* = 14) isolates were susceptible to ciprofloxacin. None of the strains were found to be universally susceptible to all the tested antimicrobials. However, large number of the isolates showed intermediate susceptible/resistance status as 92.3% (*n* = 24) to azithromycin, 65.4% (*n* = 17) to erythromycin, 65.4% (*n* = 17) to amoxicillin, 53.9% (*n* = 14) to tetracycline, 38.5% (*n* = 10) to ciprofloxacin, 34.6% (*n* = 9) to gentamycin and 34.6 % (*n* = 9) to ceftriaxone. Remarkably, a significant number of strains were fully resistant to tetracycline (46.2%, *n* = 12), amoxicillin (34.6%, *n* = 9), streptomycin (34.6%, *n* = 9), erythromycin (34.6%, *n* = 9), azithromycin (7.7%, *n* = 2) and ciprofloxacin (7.7%, *n* = 2) (Figure 3).

#### 3.4.2 Antimicrobial resistance status

Among the 26 strains of *C. fetus*, only 7.7% (*n* = 2) demonstrated full resistance against a single antimicrobial agent, AMX or ERY or AZM, while 7.7% (*n* = 2), and 19.2% (*n* = 5) exhibited resistance against two antimicrobial agents, AMX‐TET and AMX‐STR, respectively. However, 11.5% (*n* = 3) and 23.1% (*n* = 6) of the *C. fetus* strains were found to be resistant to three antimicrobial agents in two combinations: TE‐STR–AMX and STR‐ERY‐CIP, respectively. Alarmingly, as multidrug resistant, a 7.7% (*n* =2) of the *C. fetus* strains showed resistance either four (AMX‐TET‐AZM‐CTR) or five (AMX‐TET‐STR‐ERY‐AZM) antimicrobial agents in both cases. Overall, 50% (*n* = 13) strains of *C. fetus* could be identified as multidrug resistant (MDR), since these strains demonstrated resistance against three or more antimicrobial agents (Table 5).

**TABLE 5:** Distribution of antimicrobial resistance pattern among the isolated *C. fetus* (*n* = 26) strains from bulls

Resistance category	Pattern of resistance	No. (%) of strains	Subtotal [*n*, (%)]
Against one to two antimicrobial agents			
One	AMX	2 (7.7)	13 (50)
	ERY	2 (7.7)	
	AZM	2 (7.7)	
Two	TET‐AMX	2 (7.7)	
	STR‐AMX	5 (19.2)	
Against three or more antimicrobial agents (multidrug resistant)			
Three	TET‐STR‐AMX	3 (11.5)	13 (50)
	STR‐ERY‐CIP	6 (23.1)	
Four	AMX‐TET‐AZM‐CTR	2 (7.7)	
Five	AMX‐TET‐STR‐ERY‐AZM	2 (7.7)	

Abbreviations: AMX, amoxicillin; TET, tetracycline; GEN, gentamicin; ST, streptomycin; ERY, erythromycin; AZM, azithromycin; CIP, ciprofloxacin; CTR, ceftriaxone.

## 4 DISCUSSION

In this study, we evaluated animal‐level occurrence, molecular traits and antimicrobial resistance (AMR) status of *C. fetus* isolated from four breeding bull farms of Mymensingh district in Bangladesh. The study further estimated potential risk factors for the occurrence of *C. fetus* in individual breeding bulls. The study confirmed the occurrence of *C. fetus* in 75% (3/4) farms with an estimated animal/sample‐level prevalence of 8.7% (26/300). Natural service was significantly associated with *C. fetus* positive status of breeding bulls in these selected farms. The study recommends that annual screening of *C. fetus* in the breeding bulls can lead to reduced transmission in reproductive female cattle for a profitable dairy farming in Bangladesh.

This is the study for the first study of *C. fetus* of breeding bulls in Bangladesh. Since there is no published report on the occurrence of *C. fetus* in bull samples in Bangladesh, the study findings provide an initial estimate and baseline. The apparent prevalence of *C. fetus* occurrence in bull samples in this study is comparable with results from other countries. The bacterial prevalence was reported to be 8%–26% in samples collected from Brazil in cows, fetuses and bulls (de Oliveira Filho et al., 2018; Stynen et al., 2003; Ziech et al., 2014), 16.4% of bulls in Nigeria (Mai et al., 2013), 10%–15% of cows in Malawi (Klastrup & Halliwell, 1977), and 6% in India from cattle faecal samples (Mshelia et al., 2010). A lower‐level prevalence of *C. fetus*, ranging 1%–5% of samples from bulls and cows, was reported for some herds in Brazil, Argentina and Canada (Campero et al., 2017; Devenish et al., 2005; de Oliveira et al., 2015; Molina et al., 2013). However, a higher‐level prevalence of *C. fetus* was also documented in cattle in Brazil (Miranda, 2005; Pellegrin et al., 2002; Rocha et al., 2009). A study conducted in India confirmed a high prevalence of *C. fetus* positive status as 47·6%, 3·3% and 25% from of vaginal swabs, preputial wash and cervicovaginal mucous samples, respectively collected from cows and bulls (Ishtifaq et al., 2020). The reported variations in *Campylobacter* occurrence in different countries are likely related to diverse farm management practices (de Oliveira et al., 2015).

Additionally, among 37 isolates of *Campylobacter* spp., 11 (29.7%) were confirmed as *C. jejuni* from bull preputial smegma samples in the present study. A previous study in Bangladesh reported an estimated overall prevalence of *Campylobacter* spp. of 31% based on faecal samples collected from cattle, of which 70.1% were *C. jejuni* isolates (Hoque et al., 2021). The ubiquitous distribution of *C. jejuni* in the cattle faeces and farm settings may also facilitate the contamination of this pathogen in bulls’ preputial area in the farming condition in Bangladesh.

Previous investigations have claimed that exotic breeds (*Bos taurus*) are 4.2 times more likely to be susceptible to BGC, than indigenous cattle (Mai et al., 2013). However, our study has observed a significantly higher prevalence of *C. fetus* in bull herds comprising indigenous cattle (100% positive herd) than exotic breeds. The indigenous cattle in Bangladesh are frequently being subjected to natural breeding program, which may bring more likelihood of *C. fetus*‐positive status through exposure of infection from the infected cows during coitus.

Natural breeding scheme is considered as the main contributing factor for the maintenance of *C. fetus* in the herd (BonDurant, 2005). Use of natural breeding program rather than artificial insemination was found to be important risk factor BGC in the present study. Nevertheless, C. *fetus* was detected for one of the two larger farms in our study (Figure 2, Farm A and D) known to be practicing commercial artificial insemination (AI) breeding program with annual screening for *C. fetus* in the bull herds. Frequent infection through artificial insemination can happen in the territories where microbiological screening specifically targeting *C. fetus* is not well addressed or strictly adhered to (Irons et al., 2004; World Organization for Animal Health, 2018). Additionally, history of venereal disease in animal and mounting behaviour (mounting to other animals) would be potential risk factors of *C. fetus* occurrence in bulls, also noted (Taylor, 2002); however, these were found to be insignificant in this study.

Increasing incidence of antimicrobial resistance in *Campylobacter* spp. is a global concern. The World Health Organization (WHO) has listed of multidrug‐resistant (MDR) bacteria as a significant public health burden (Centers for Disease Control and Prevention, 2010; World Health Organization, 2001; Ethelberg et al., 2005). In Bangladesh, *C. jejuni* and *C. coli* being isolated from farmed animals, predominantly poultry, have been reported by a number of studies (Alam et al., 2020; Islam et al., 2018; Kabir et al., 2014; Neogi et al., 2020). However, systematic investigations observing antimicrobial resistance in *C. fetus* strains isolated from bull herds have so far received little focus. In this regard, data obtained in the present study can be considered as useful base‐line information. Resistance to tetracycline, amoxicillin and streptomycin among a significant portion of *C. fetus* strains obtained from the bull smegma specimens was 6.2%, 34.6% and 34.6%, respectively, is a concern. Moreover, nearly half of the *C. fetus* strains (46.1%) were resistant to ciprofloxacin, and nearly one‐third (34.6%) of these strains were also found to be resistant to gentamicin. Similar higher occurrence of resistance to these antimicrobial agents has been also reported in *C. jejuni* and *C. coli* in poultry from Bangladesh (Alam et al., 2020; Islam et al., 2018; Kabir et al., 2014; Neogi et al., 2020). A study in Canada revealed that *C. fetus* isolates in faecal samples had substantial resistance to tetracycline, doxycycline and ciprofloxacin (Inglis et al., 2006). Interestingly, the majority of the *C. fetus* strains in this study were observed to exhibit intermediate resistance to azithromycin (92.3%), followed by erythromycin (65.4%), amoxicillin (65.4%) and tetracycline (53.9%) with a variable degree of susceptibility to other antimicrobials. These results can be linked to the large‐scale application of antimicrobials in animal farms for both prophylactic and therapeutic purposes (Chowdhury et al., 2009). In comparison to previously documented observations for *C. jejuni* and *C. coli* strains isolated from animal farms in Bangladesh (Alam et al., 2020; Neogi et al., 2020), occurrence of this kind of intermediate‐resistant strains is relatively higher among the *C. fetus* strains of the present study. A previous study has reported an abundance of intermediate‐resistant strains in live bird markets with multiple species than poultry farms, which can be attributable to natural decay of active component of antimicrobials (Neogi et al., 2020). Antimicrobial residues, at sublethal concentration, may modulate the bacterial genetic mechanisms conferring intermediate resistance and MDR traits in *Campylobacter* strains. Inter‐ and intra‐species horizontal transfer of multiple‐resistant genes, through mobile genetic elements (e.g., plasmids, Class I integron, conjugative transposon) and mutations in genes regulating efflux pumps, for example, *gyrA* and *gyrB* of DNA gyrase and *parC* and *pare* of topoisomerase IV, are the known genetic mechanisms associated with antimicrobial resistance in *Campylobacter* spp. (Hoshino et al., 1998). A remarkable observation of the present study is the high incidence of MDR in *C. fetus* (50% strains) in bull samples, demonstrating co‐resistance against three (TET‐STR‐AMX and STR‐ERY‐CIP), four (AMX‐TET‐AZM‐CTR) and even five (AMX‐TET‐STR‐ERY‐AZM) antimicrobial agents. A variable distribution (34.9%–83.36% and 30%–100% for *C. jejuni* and *C. coli*, respectivel*y*) of MDR in *Campylobacter* spp. has been reported for poultry in Bangladesh (Alam et al., 2020; Kabir et al., 2014; Neogi et al., 2020). The observed resistant patterns from the current study could be considered as serious threat to public health since *Campylobacter* spp. are important zoonotic pathogens, mostly associated with foodborne infections. Despite government efforts to control antimicrobial resistance in animal husbandry, including restricted use for therapeutic purposes only, the majority the farmers in this country are applying a variety of antimicrobial agents as feed additives to enhance meat production. Farmers are not following appropriate guidelines, including suggestions on the withdrawal period for specific antimicrobials, from a registered veterinarian in Bangladesh (Department of Livestock Services, 2010). Notwithstanding, global declaration to minimise AMR and preserve the efficiency of antimicrobials are crucial; however, developing countries have not yet been successful in mainstreaming such strategies to significantly lessen the overuse and or even misuse of antimicrobial agents (McGettigan & Henry, 2011). Therefore, mass‐scale participatory efforts and incentives are needed to minimise the use antimicrobials in food animal production under a One Health platform, including human health, animal health and environmental health perspectives.

The information from this study are beneficial for systematic evaluation of *C. fetus* infections, including BGC, transmission among cattle herds and accompanying economic losses in Bangladesh or even other low‐resource countries where reproductive natural breeding programs are most common (Garcia, 2009; Lage & Leite, 2000). The findings of this study support evidence‐based decision‐making to prioritise annual/seasonal screening of breeding bulls for *C. fetus* contamination to confirm breeding soundness. Infected bulls should be segregated from herds; however, a culling option requires a compensatory financing mechanism where resource constraints currently exist.

This is also an inherent limitation of a cross‐sectional study design since exposure and outcome variables are collected simultaneously. However, more information on additional risk factors could be obtained from case‐control (outcome is recorded first and the frequency of exposure recorded) or cohort studies (exposure is recorded first and followed for the outcome). Moreover, multi‐locus sequence typing, differentiation into subspecies and phylogenetic analysis to estimate clonal relatedness would be required for a comprehensive understanding of the diversity, dynamics and source tracking of *C. fetus* populations circulating in the cattle herds. Therefore, future studies with in‐depth molecular analysis to estimate genomic diversity in *C. fetus* strains to be obtained by representative sampling from herds and animals in Bangladesh are intended and suggested.

## 5 CONCLUSIONS

The occurrence of *C. fetus* in preputial smegma samples of breeding bulls indicates that bulls can act as a potential reservoirs for the transmission of this pathogen to healthy female cattle in Bangladesh. Therefore, annual screening of bulls for evaluation of breeding soundness should be mandatory. The coverage of artificial insemination with *C. fetus* infection free bulls’ semen can be broaden to minimise reproductive failures and support to the livelihood of marginal dairy farmers. Moreover, the large‐scale occurrence of multidrug‐resistant strains of *C. fetus* in bull samples in this study highlights the clear need to implement the judicial use of antimicrobial agents in veterinary treatment to protect for both animal and human health. Finally, the results of this study indicate the critical need to develop and implement official guidelines related to breeding bull screening, culling and segregation of infected animals to reduce *C. fetus* infection in bull herds and support to livestock production and food security in Bangladesh.

## CONFLICT OF INTEREST

The authors declare that they have no competing interest exists regarding financial or personal that could have appeared to influence the work reported in this paper.

## AUTHOR CONTRIBUTIONS

Nazmul Hoque: data curation; formal analysis; investigation; methodology; writing – original draft. Sk Shaheenur Islam: formal analysis; writing – original draft. Mahmudul Hasan Sikder: writing – review & editing. S. M. Lutful Kabir: conceptualisation; writing – review & editing.

## ETHICAL APPROVAL AND INFORMED CONSENTS

The Animal Welfare and Experimentation Ethical Committee (AWEEC) of Bangladesh Agricultural University approved the study under the sanction no. AWEEC/BAU/2019(45). A verbal permission was taken from the owner/manager of the cattle farms during data collection and subsequent animal sampling. Animals were handled humanely to lessen suffering.

### PEER REVIEW

The peer review history for this article is available at https://publons.com/publon/10.1002/vms3.831

## Supporting information

Supporting Information

Supporting Information

## Data Availability

The data that support the findings of this study are included in the manuscript.
