# Supplementary figures and images for: Investigation of Campylobacter fetus in breeding bulls of private farms in Bangladesh
Source: Vet Med Sci. 2022 Jul 11;9(1):417–28. doi: 10.1002/vms3.831 (PMC9857011; doi:10.1002/vms3.831)

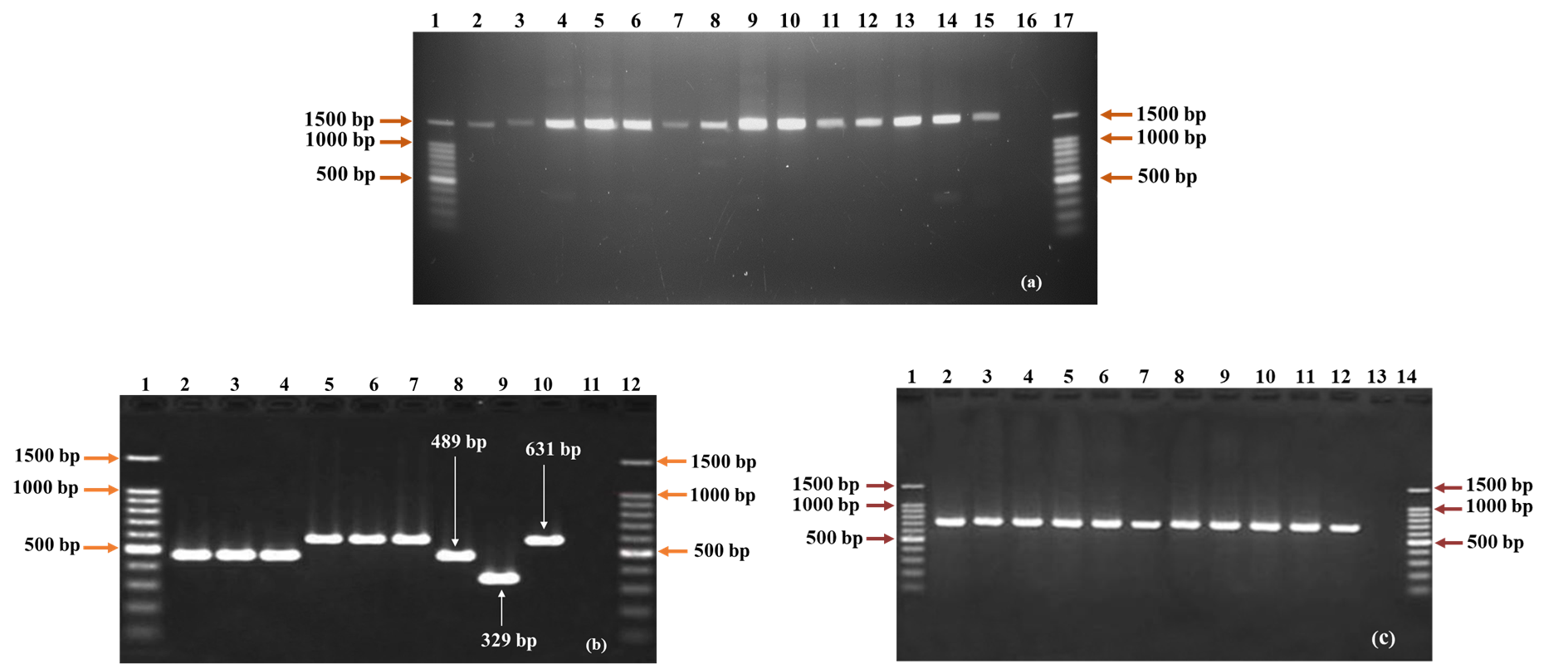

Supplement: Supplementary file 1 — Supporting Information [file VMS3-9-417-s001.tif]
