# Supplementary material for: Investigation of Campylobacter fetus in breeding bulls of private farms in Bangladesh
Source: Vet Med Sci. 2022 Jul 11;9(1):417–28. doi: 10.1002/vms3.831 (PMC9857011; doi:10.1002/vms3.831)
Supplement: Supplementary file 2 — Supporting Information [file VMS3-9-417-s002.docx]

**Supplementary Table S1: Questionnaire on assessment of herd level and animal level risk factors for *Campylobacter fetus* in bull farms of Mymensingh districts in Bangladesh**

| **Questionnaire ID:………** | |  |  |  |  |  |  | |  |  |
| --- | --- | --- | --- | --- | --- | --- | --- | --- | --- | --- |
| **Date of interview: …./…../20…….** | | |  |  |  |  |  | |  |  |
| **Section A: Herd composition and management practices** | | | | | | | | | |  |
| **1. Location of the farms** | | |  |  |  |  |  | |  |  |
| Village:……… | | Upazila:…….. | | | District: | |  | |  |  |
|  |  |  |  |  | - Dhaka | Mymensingh | | | |  |
| **2. Breed** | |  |  |  |  |  |  | |  |  |
|  | - Crossbred | - Indigenous | | |  |  |  | |  |  |
| **3. Type of husbandry** | |  |  |  | **4. Herd size** | |  | |  |  |
|  |  |  |  |  |  |  |  | |  |  |
| - Intensive | | |  |  | - 10-20 | |  | |  |  |
| - Extensive/semi-intensive | | |  |  | - 20-50 | |  | |  |  |
|  |  |  |  |  | - >50 | |  | |  |  |
| **5. Biosecurity status** | | | |  | **6. Keeping other animals in the farm (sheep and goat)** | | |  | |  |
|  |  |  |  |  |  |  |  | |  |  |
| - Good | | |  |  |  | - Yes | | | | |
| - Bad | | |  |  |  | - No | | | | |
|  | |  |  |  |  |  |  | |  |  |
| **7. Breeding bull use type** | | |  |  | **8. Presence of biogas plant** | | | | |  |
| - Natural | | |  |  |  | - Yes |  | |  |  |
| - Artificial | |  |  |  |  | - No |  | |  |  |
| **9. Routine testing of Campylobacter fetus in the herd** | | | | | **10. Feed** | | |  | |  |
|  |  |  |  |  |  |  |  | |  |  |
| - Yes |  |  |  |  | - Commercial (TMR) | |  | |  |  |
| - No |  |  |  |  | - Ready-made feed | |  | |  |  |
|  |  |  |  |  |  |  |  | |  |  |
|  |  |  |  |  |  |  |  | |  |  |

| **Section B: Animal level risk factor** | | | | | | | | | | | | |
| --- | --- | --- | --- | --- | --- | --- | --- | --- | --- | --- | --- | --- |
| **1. Age of the animal** | | | | | | | | | | |  | |
|  |  |  | |  |  |  | |  | |  | |  |
| - >3 years | | |  | |  |  |  | |  | |  | |
| - 3years | | |  | |  |  |  | |  | |  | |
|  |  |  | |  |  |  | |  | |  | |  |
| **2. Weight of the animal** | | |  | | **3. Breed** | | | | | | | |
|  | - Up to 300 kg |  | |  |  | - Indigenous | |  | |  | |  |
|  | - Above 300 kg |  | |  |  | - Crossbred | |  | |  | |  |
|  |  |  | |  |  |  | |  | |  | |  |
| **4. Breeding bull use type** | | | | | | **5. History venereal disease** | | |  | |  | |
|  |  |  | |  |  |  | |  | |  | |  |
|  | - Natural service | | |  |  | - Yes | |  | |  | |  |
|  | - Artificial insemination | | |  |  | - No | |  | |  | |  |
| **6. Bad behavior (Mounting)** | |  | |  | **7.** | **Animal level Status *(C. fetus)*** | | | | | | |
|  | - Yes |  | |  |  | - Positive | |  | |  | |  |
|  | - No | |  | |  | - Negative |  | |  | |  | |
|  |  |  | |  |  |  | |  | |  | |  |

**Supplementary FIGURE S1.** Molecular detection: (a) confirmation of *Campylobacter* spp. by 16S rRNA gene, lanes 1 and 17: 100 bp DNA ladder (Promega, USA), lanes 2–12: representative positive isolates, lane 13: positive control (*C. fetus* ATCC 27374), lane 14: positive control (*C. coli* ATCC 33559), lane 15: positive control (*C. jejuni* ATCC 33560), and lane 16: negative control (*Escherichia coli* ATCC 25922); (b) confirmation of *C. fetus* and *C. jejuni* via *cdtA* gene-based multiplex PCR assay, lanes 1 and 12: 100 bp DNA ladder (Promega, USA), lanes 2–4: representative positive isolates (*C. fetus*), lanes 5–7: representative positive isolates (*C. jejuni*), lane 8: positive control (*C. fetus* ATCC 27374), lane 9: positive control (*C. coli* ATCC 33559), lane 10: positive control (*C. jejuni* ATCC 33560), and lane 11: negative control (*Escherichia coli* ATCC25922) and (c) validation of *C. jejuni* by hippuricase (*hipO*) gene-based PCR, lanes 1 and 14: 100 bp DNA ladder (Promega, USA), lanes 2–11: representative positive isolates, lane 12: positive control (*C. jejuni* ATCC 33560), lane 13: negative control (*Escherichia coli* ATCC 25922).
